# Stereoselective Regulation of P-gp Activity by Clausenamide Enantiomers in Caco-2, KB/KBv and Brain Microvessel Endothelial Cells

**DOI:** 10.1371/journal.pone.0135866

**Published:** 2015-08-21

**Authors:** Chuan-jiang Zhu, Fang Hua, Xiao-lu Zhu, Meng Li, Hong-xu Wang, Xiao-ming Yu, Yan Li

**Affiliations:** 1 Institute of Materia Medica, Chinese Academy of Medical Sciences & Peking Union Medical College, Beijing, China; 2 Beijing Key Laboratory of Non-Clinical Drug Metabolism and PK/PD Study, Beijing, China; 3 Beijing Key Laboratory of New Drug Mechanisms and Pharmacological Evaluation Study, Beijing, China; 4 Beijing Key Laboratory of Active Substance Discovery and Druggability Evaluation, Beijing, China; The University of Iowa, UNITED STATES

## Abstract

The (−)- and (+)-clausenamide (CLA) enantiomers have different pharmacokinetic effects in animals, but their association with putative stereoselective regulation of P-glycoprotein (P-gp) remains unclear. Using three cells expressing P-gp—Caco-2, KBv and rat brain microvessel endothelial cells(RBMEC), this study investigated the association of CLA enantiomers with P-gp. The results showed that the rhodamine 123 (Rh123) accumulation, an indicator of P-gp activity, in Caco-2, KBv and RBMECs was increased by (−)CLA (1 or 5 μmol/L) at 8.2%–28.5%, but reduced by (+)CLA at 11.7%–25.9%, showing stereoselectivity in their regulation of P-gp activity. Following co-treatment of these cells with each CLA enantiomer and verapamil as a P-gp inhibitor, the (+)-isomer clearly antagonized the inhibitory effects of verapamil on P-gp efflux, whereas the (−)-isomer had slightly synergistic or additive effects. When higher concentrations (5 or 10 μmol/L) of CLA enantiomers were added, the stimulatory effects of the (+)-isomer were converted into inhibitory ones, leading to an enhanced intracellular uptake of Rh123 by 24.5%–58.2%; but (−)-isomer kept its inhibition to P-gp activity, causing 30.0%–63.0% increase in the Rh123 uptake. The biphasic effects of (+)CLA were confirmed by CLA uptake in the Caco-2 cells. (+)CLA at 1 μmol/L had significantly lower intracellular uptake than (−)CLA with a ratio[(−)/(+)] of 2.593, which was decreased to 2.167 and 1.893 after CLA concentrations increased to 2.5 and 5 μmol/L. Besides, in the non-induced KB cells, (+)CLA(5 μmol/L) upregulated P-gp expression at 54.5% relative to vehicle control, and decreased Rh123 accumulation by 28.2%, while (−)CLA(5 μmol/L) downregulated P-gp expression at 15.9% and increased Rh123 accumulation by 18.0%. These results suggested that (−)CLA could be a P-gp inhibitor and (+)CLA could be a modulator with concentration-dependent biphasic effects on P-gp activity, which may result in drug—drug interactions when combined with other P-gp substrate drugs.

## Introduction

P-glycoprotein(P-gp) belongs to a superfamily of ATP binding cassette transport proteins and plays the role of an energy-dependent drug efflux pump[1,2]. It is expressed in tumour cells (*e*.*g*. vinblastine-treated human KB carcinoma cell lines) resulting in multiple drug resistance[3,4], and in epithelial cells(*e*.*g*. intestine and Caco-2) and endothelial cells(*e*.*g*. brain microvessels on the blood—brain barrier) leading to decreased bioavailability of its substrates[1,3–7]. Rhodamine 123(Rh123) is a fluorescent P-gp substrate and is often used to study P-gp activity and to evaluate the relationship of candidate drugs with P-gp(*e*.*g*. substrate, inhibitor, inducer, or modulator) [1,3,5,7,8].

3S, 4R, 5R, 6S-clausenamide [(−)CLA] is a eutomer and candidate drug currently being developed to treat Alzheimer’s disease, but its antipode, 3R,4S,5S,6R-CLA [(+)CLA], is a distomer[9–15]. Pharmacokinetic studies showed that there was a significant stereoselective difference in AUC_0–12 h_ values 1256 ± 308 min·μg/mL (n = 6) for (−)CLA and 2446 ± 540 min·μg/mL(n = 6; *P* <0.001) for (+)CLA after oral administration to rats[16]. Furthermore, studies on the first-pass metabolism of CLA enantiomers following an oral dose of 100 mg/kg in rabbits showed that (−)CLA had a smaller AUC_0–8 h_(1001 ± 487 h·μg/mL, n = 5) than did (+)CLA(2453 ± 1101 h·μg/mL, n = 4; *P* < 0.01) in the portal vein[17]. Moreover, distributional kinetics in target tissues (*e*.*g*. hippocampus, cortex and cerebellum) showed that (+)CLA had greater values for AUC_0–∞_ than did (−)CLA [18]. These data suggest that the absorptive and distributional processes of CLA enantiomers could be involved in P-gp in animal intestines and brain microvessels.

Therefore, this study used three cells expressing P-gp, Caco-2, KBv and rat brain microvessel endothelial cells (RBMEC), to investigate the interaction of P-gp with CLA enantiomers. The results from this study indicated that the (−)-isomer could be a P-gp inhibitor, but the (+)-isomer could be a modulator with concentration-dependent biphasic effects and showed stereoselective regulation of P-gp activity and possible drug—drug interactions when combined with other P-gp substrate drugs.

## Materials and Methods

### Chemicals and Reagents

(-)CLA and (+)CLA were synthesized respectively and supplied kindly by Prof. L. Huang’s laboratory in our institute[11,16] and Guangzhou Nuohao Medical Technology Co., Ltd(Guangzhou, China). The optical purity of each CLA isomer was over 99%. Human epithelial colorectal adenocarcinoma cell (Caco-2, passage 32) was obtained from Cell Resource Center, Peking Union Medical College (Beijing, China). Human KB carcinoma cell line (KB) and vinblastine-resistant KB(KBv) were kindly provided by Dr. H. Sun in our institute[19]. Tissue culture plates(6-well) were obtained from Corning Costar(Cambrige, MA, USA). Fetal bovine serum(FBS), minimum essential medium(MEM), RPMI 1680 medium, and non-essential amino acids(NEAA) were purchased from Gibco-Invitrogen Corporation (Grand Island, New York, USA). Rhodamine123 (Rh123), bovine serum albumin (BSA) were obtained from Sigma-Aldrich Chemical Co.(St. Louis, MO, USA). Verapamil hydrochloride, cyclosporine A, and glipizide were purchased from National Institutes for Food and Drug Control(Beijing, China). The mouse monoclonal antibody raised against amino acids of 1040–1280 of P-gp was purchased from Santa Cruz Biotechnology(Santa Cruz, CA, USA). The mouse anti-GAPDH monoclonal antibody, peroxidase-conjugated goat anti-mouse IgG secondary antibody, were from Beijing Zhongshan Golden Bridge Biotechnology Co., Ltd(Beijing, China). Rabbit anti-factor VIII polyclonal IgG, and FITC-conjugated goat anti-rabbit factor VIII secondary antibody were from Beijing Biosynthesis Biotechnology Co., Ltd(Beijing, China). Immobilon-P membranes were from Millipore Corporation(Bedford, MA, USA) and Western luminescent detection kit was from Vigorous Biotechnology Beijing Co., Ltd(Beijing, China). All other chemicals and reagents were of the highest quality commercially available.

### Rh123 accumulation in Caco-2 cells

Caco-2 cells were seeded onto 6-well plates at a density of 5 × 10^5^ cells/well and maintained in MEM supplemented with 10% (v/v) FBS, 1% (v/v) NEAA, 100 units/mL penicillin and 100 μg/mL streptomycin sulphate in a humidified 37°C incubator with 5% CO_2_. Upon 80%–90% confluence, the cells were provided new complete medium (2 mL/well) and exposed, to vehicle, (−)CLA (1 or 5 μmol/L), (+)CLA (1 or 5 μmol/L), verapamil (200 μmol/L) or individual enantiomer (1 or 5 μmol/L) plus verapamil (200 μmol/L). Then, Rh123 (5 μmol/L) was added to all the wells. After incubation at 37°C and 5% CO_2_ for 2 h which was designed by the pharmacokinetic parameters of CLA enantiomers[16], the cells were washed 3 times with 2 mL of ice-cold PBS (pH 7.4), lysed in 400 μL of lysis buffer [50 mmol/L Tris-HCl (pH 8.0), 150 mmol/L NaCl, 0.1% SDS, 100 μg/mL PMSF, 1 μg/mL aprotinin and 1% Nonidet P-40], and then were placed on ice for 40 min. The plates were wrapped in tinfoil. The fluorescence of Rh123 in each well was detected by Spectra Max Gemini XS plate reader (Molecular Devices Corp., Sunnyvale, CA, USA) with excitation and emission wavelengths of 488 nm and 530 nm, respectively. Meanwhile, 10 μL of cell lysates were collected from each well to measure protein concentration using Lowry’s method[20]. Intracellular Rh123 accumulation was normalized by protein concentration (mg/mL) and then was calculated by a standard curve of Rh123 at three concentrations (2.5–10 μmol/L), in parallel with the lysis experiment.

### Rh123 accumulation in RBMECs

The experimental programs in this part were approved by the Animal Care and Welfare Committee in our institute. Animals were treated in accordance with national/international guidelines. Primary cultures of brain microvessel endothelial cells were prepared from Sprague—Dawley rats (10–12 days old; Beijing Vital River Company, Beijing, China), as previously described by Qian *et al*.[21]. The gray matter of animals was taken out and minced into small pieces of approximately 1 mm^3^ in ice-cold MEM medium, then dissociated by using 2 mg/mL collagenase containing DNase at a final concentration of 10.5 μg/ml on a shaker for 20 min at 37°C. The cells were pelleted by centrifugation(1000 rpm, 10 min, 4°C) and digested once again according to the above protocol. Then 20% dextran(Mr 40,000) was added to the cells for differential centrifugation at 2000 rpm for 10 min. The cells were re-suspended in complete MEM medium and passed through 74-μm stainless steel mesh. Obtained RBMECs were seeded on 1% (w/v) gelatin-coated culture flasks and cultivated in complete medium (MEM, 20% FBS, 0.9 mg/mL glutamate, 10 mmol/L hepes, 100 ng/mL bFGF, 100 μg/mL heparin sulphate, 100 units/mL penicillin, 100 μg/mL streptomycin sulphate and 50 units/mL amphotericin B) at 37°C and 5% CO_2_, which was refreshed with complete medium 4 h after seeding to eliminate non-endothelial cells. A final concentration of 2 μg/mL of puromycin was added for 7 h to further purify the cultured cells. The purity of RBMECs was examined by their morphological characteristics using a phase-contrast microscope, and immunocytochemistry of a specific marker, factor VIII-associated antigen, with a rabbit anti-factor VIII polyclonal antibody and FITC-conjugated goat anti-rabbit factor VIII secondary antibody by immunofluorescence microscopy (Olympus IX 70-S1Z2, Olympus Optical CO. Ltd., Japan). The P-gp expression in cultured cells was determined by Western blot analyses using a mouse monoclonal antibody raised against amino acids 1040–1280 of P-gp. When the cultures in the flasks reached 80%–90% confluence, the purified RBMECs were passaged by a brief treatment with trypsin (0.25%, w/v) and EDTA (0.02%, w/v) solution and seeded onto 6-well plates at a density of 5 × 10^5^ cells/well; after 2–3 days, incubated with the following agents for 2 h under culture conditions (37°C, 5% CO_2_ and humidified air): vehicle; (−)CLA (1 or 5 μmol/L); (+)CLA (1 or 5 μmol/L); verapamil (200 μmol/L); or individual enantiomer (1 or 5 μmol/L) plus verapamil (200 μmol/L). In the end, Rh123 (5 μmol/L) was added to the whole wells. The cell incubation, solubilisation and Rh123 accumulation were performed according to the same method for Caco-2 cells described above.

### Rh123 accumulation in KB/KBv cells

KB or its vinblastine-resistance derivative KBv cells[19] were maintained on 6-well plates (5 × 10^5^ cells/well) in RPMI 1680 medium with 10% FBS, 100 units/mL penicillin and 100 μg/mL streptomycin sulphate at 37°C with 95% air and 5% CO_2_. After reaching 80%–90% confluence, the two cells were treated with the following agents, respectively: vehicle; (−)CLA (5 or 10 μmol/L); (+)CLA (5 or 10 μmol/L). Additionally, the KBv cells were treated with verapamil (200 μmol/L); or individual enantiomer (5 or 10 μmol/L) plus verapamil (200 μmol/L). Rh123 (5 μmol/L) were finally added to these wells. Subsequently, cell incubation, solubilisation and Rh123 accumulation were performed by using the same method for Caco-2 cells.

### P-gp expression in KB cells: Western blots

The sensitive, non-induced KB cells on 6-well plates were grown to 80%–90% confluence and incubated with the following agents for 24 h at 37°C and 5% CO_2_: vehicle, (−)CLA (1 or 5 μmol/L), (+)CLA (1 or 5 μmol/L), or verapamil (100 μmol/L). The cells were washed 3 times with ice-cold PBS (pH 7.4) and were lysed with 80 μL of lysis buffer described above. Lysates were frozen with liquid nitrogen, thawed in water at 37°C three times and centrifuged at 15,000 ×*g* for 10 min. The protein concentration of the supernatant was determined by Lowry’s method with bovine serum albumin as the standard. Proteins (48 μg) were separated by SDS-PAGE on a 10% separating gel and 4% stacking gel, and were transferred onto Immobilon-P membranes. The transferred proteins were treated with a blocking buffer (5% non-fat dried milk and 0.02% NP-40 in PBS) and then incubated with P-gp or GAPDH antibodies (at a 1:100 dilution) or HRP-conjugated secondary antibodies (1:1000 dilution) at 37°C for 1 h, respectively. The protein bands were visualized with enhanced chemiluminescence according to the manufacturer’s instructions. The density of the bands was quantified by scanning densitometry (LAS-3000 Imaging System, Fujifilm, Japan; and Kodak Digital Science 1D Analysis Software, Rochester, NY, USA) and was expressed as normalized values to GAPDH.

### Uptake of CLA enantiomers in Caco-2 cells

Caco-2 cells on 6-well plates (5 × 10^5^ cells/well) were cultured in MEM containing 10% (v/v) FBS, 1% (v/v) NEAA, 100 units/mL penicillin and 100 μg/mL streptomycin sulphate at 37°C with 95% air and 5% CO_2_. When 80%–90% confluence was observed using an inverted light microscope, the cells were treated, respectively, with (−)CLA or (+)CLA at 1, 2.5 and 5 μmol/L for 2 h, washed 3 times with ice-cold PBS, collected into 1.5-mL Eppendorf tubes and centrifuged at 1500 rpm for 5 min. The precipitates were lysed with 80 μL of lysis buffer described above, frozen with liquid nitrogen, then thawed in water at 37°C three times, and then centrifuged at 10,000 rpm for 10 min (4°C). We used 10 μL of supernatant for the determination of protein concentration using Lowry’s method with bovine serum albumin as the standard, and the residue of the supernatants was subjected to extraction with ethyl acetate. The internal standard (IS) was 20 μL of glipizide (5.2 μg/mL). Extracted CLA enantiomer and glipizide were measured by a liquid chromatography-tandem mass spectrometr(LC-MS/MS).

The LC-MS/MS system consisted of an Alliance HT 2795 LC pump, an autosampler, and a Micromass Quattro Premier tandem quadrupole mass spectrometer with an electrospray ionization (ESI) source and Masslynx version 4.1 software (Waters Corp., Milford, MA, USA). The separation of CLA enantiomer and glipizide was performed using an XBridge C18 column (2.1 mm × 50 mm; i.d., 3.5 μm) (Waters Corp., Milford, MA, USA). The column temperature was maintained at 22°C, and the sample injection volume was 20 μL in the autosampler at 4°C. Mobile phases A and B were water with 0.1% (v/v) acetic acid and acetonitrile with 0.1% (v/v) acetic acid, respectively. Gradient elution was used at a flow rate of 0.25 mL/min for 8 min with 80% A and 20% B for 0–2 min, 5% A and 95% B for 2–4 min and 80% A and 20% B for 4.01–8 min. The LC system was connected to the mass spectrometer by an electrospray interface. The positive-ion ESI mode was selected, and the electrospray capillary voltage was 3.5 kV. Desolvation gas (ultrapure nitrogen) flow and cone gas flow were provided separately at rates of 600 L/h and 50 L/h, and source and desolvation temperatures were set at 110°C and 450°C, respectively. Argon was used as a collision gas in collision-induced dissociation of precursor ions at a flow rate of 0.27 mL/min. The data were acquired using multiple reaction monitoring mode and analysed by Masslynx software 4.1. The main parameters (retention time, ion channel, cone voltage and collision energy) were as follows: 2.66 min, 298.2–174.1, 25 V and 17 V for CLA enantiomer; and 2.97 min, 446.7–321.2, 25 V and 12 V for glipizide (IS), respectively. The lower limit of quantification of CLA enantiomer was 0.25 ng/mL. The mean relative recovery was 89%, and intra-assay and inter-assay coefficients of variation were <15%. The range of linearity was 0.25–200 ng/mL with *r* = 0.999. The concentrations of (−) or (+)CLA were quantified by standard curves according to their peak areas against the IS.

### Statistical analysis

Data from each experiment for measuring Rh123 intracellular accumulation were divided into two groups in pairs: vehicle control and CLA treatment, and verapamil control and co-treatment of verapamil with CLA. Their effects were displayed separately in two figures but using the same vertical axes and were assessed respectively by one-way analysis of variance followed by Student—Newman—Keuls test. Student *t*-test was used to assess the differences between two enantiomers in the induction of P-gp expression in KB cells and uptake in Caco-2 cells, respectively. All experiments were performed at least three times. Results were presented as mean values ± SD. *P* < 0.05 was considered to be statistically significant.

## Results

### Effects of CLA enantiomers on the Rh123 accumulation in Caco-2 cells

**Fig 1:**
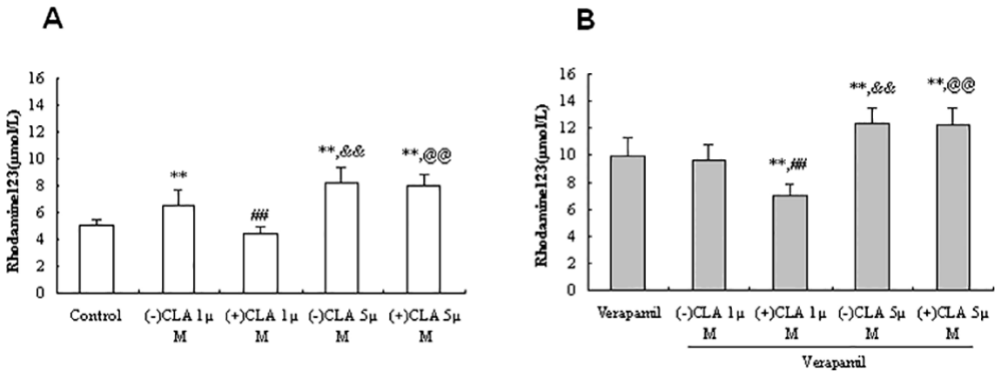
Effects of clausenamide(CLA) enantiomers at different concentrations on the accumulation of rhodamine 123 in the absence(A) or presence(B) of P-gp inhibitor verapamil in Caco-2 cells. Data were presented as Mean±SD from 6 independent experiments; (-) or (+)CLA: 1 or 5 μmol/L; verapamil: 200 μmol/L; rhodamine123: 5 μmol/L; **P<0.01 *vs*. Control or Verapamil; ^##^ *P*<0.01 *vs*. (-)CLA 1 μmol/L; ^&&^ *P*<0.01 *vs*. (-)CLA 1μmol/L; ^@@^ *P*<0.01 *vs*. (+)CLA 1μmol/L.

Treatment of Caco-2 cells with (−)CLA (1 μmol/L) elicited significantly enhanced intracellular accumulation of Rh123 by 28.5% relative to vehicle control (Fig 1A; *P* < 0.01), indicating that (−)CLA had the same inhibitory effects as verapamil (Fig 1B) on P-gp activity. On the contrary, (+)CLA decreased the Rh123 intracellular accumulation by 11.7%, showing there were significant differences in interplay with P-gp between the two enantiomers (*P* < 0.01). Furthermore, co-treatment of (+)CLA (1 μmol/L) with verapamil (200 μmol/L) resulted in significantly reducing Rh123 intracellular concentration than that following verapamil treatment alone by 29.3% (*P* < 0.01), suggesting the stimulation of (+)CLA on the P-gp efflux activity(Fig 1B). When a higher concentration (5 μmol/L) was added to the cells, the stimulatory effects of (+)CLA were converted into inhibitory ones on P-gp activity, which resulted in significantly increased Rh123 accumulation by 58.2% (*P* < 0.01 *vs*. control), near its antipode, by 63.0% (Fig 1A). Verapamil plus (−) or (+)CLA (5 μmol/L) showed synergistic effects, leading to more Rh123 accumulation by 24.2% and 23.1%, respectively, than did verapamil alone (Fig 1B; *P* < 0.01 *vs*. verapamil).

### Effects of CLA enantiomers on the Rh123 accumulation in RBMECs

**Fig 2:**
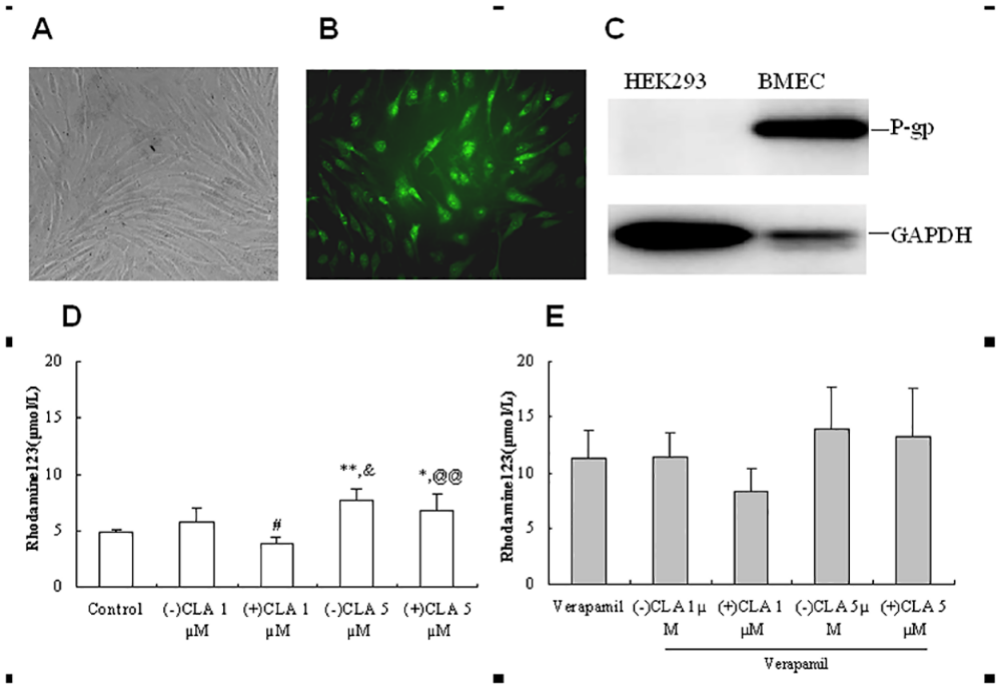
Observation of the morphology(A), specific marker (B) and P-gp expression(C) of primary rat brain microvessel endothelial cells(RBMEC), and effects of clausenamide(CLA) enantiomers at different concentrations on the accumulation of rhodamine 123 in the absence(D) or presence(E) of P-gp inhibitor verapamil in the RBMECs. Data were presented as Mean±SD from 4–5 independent experiments; (-) or (+)CLA: 1 μmol/L; rhodamine123(Rh123): 5 μmol/L; *P<0.05, **P<0.01 *vs*. Control; ^#^ *P*<0.05 *vs*. (-)CLA; ^&^ *P*<0.05 *vs*. (-)CLA 1 μmol/L; ^@@^ *P*<0.01 *vs*. (+)CLA.

The shape of primary RBMECs upon 80%–90% confluence was shown in Fig 2A. A positive fluorescent immunostaining for factor VIII-related antigen, a marker for endothelial cells (Fig 2B), and the expression of P-gp protein were also observed in the cells (Fig 2B and 2C; HEK293 cells were used as blank controls), showing their higher purity and higher expression level of P-gp. Following addition of (−) or (+)CLA to RBMECs at 1 μmol/L, the (−)-isomer increased the Rh123 accumulation by 20.0% relative to vehicle control, whereas the (+)-isomer decreased Rh123 accumulation by 20.0%, showing significantly stereoselective differences between the two enantiomers (*P* < 0.05; Fig 2D). Co-treatment of verapamil with (+)CLA (1 μmol/L) showed antagonistic effects, decreasing in Rh123 accumulation by 26.5%, but verapamil plus (−)CLA showed a slightly increased trend relative to verapamil alone (Fig 2E). When the higher concentration of 5 μmol/L of CLA enantiomer was added to the RBMECs, biphasic effects of (+)CLA were observed with a significant increase of Rh123 accumulation by 41.1% (*P* < 0.05 *vs*. control), lower than its antipode by 58.4%. Co-treatment of (−) or (+)CLA (5 μmol/L) with verapamil showed synergistic inhibition to P-gp efflux and an increase in Rh123 accumulation by 24.5% and 18.5%, respectively, relative to verapamil alone (Fig 2E).

### Effects of CLA enantiomers on the Rh123 accumulation in KBv cells

**Fig 3:**
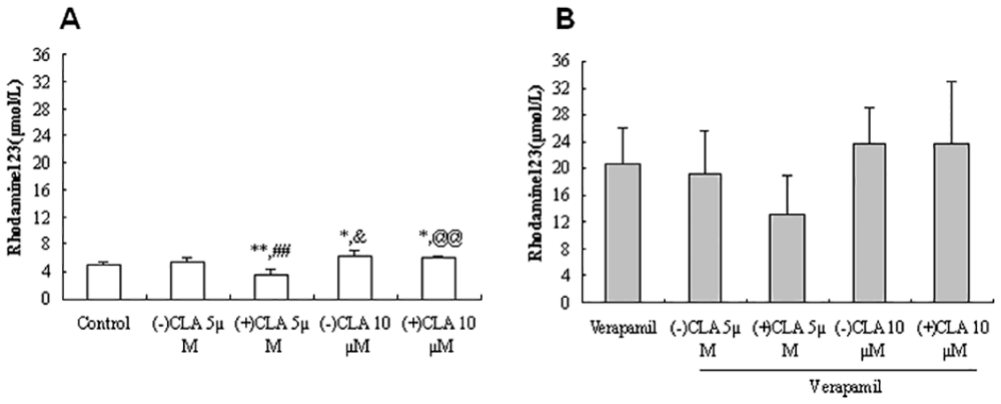
Effects of clausenamide(CLA) enantiomers at different concentrations on the accumulation of rhodamine 123 in the absence(A) or presence(B) of P-gp inhibitor verapamil in KBv cells. Data were presented as Mean±SD from 4–5 independent experiments; (-) or (+)CLA: 5 μmol/L or 10 μmol/L; verapamil: 200 μmol/L; rhodamine123: 5 μmol/L; *P<0.05, **P<0.01 *vs*. Control; ^##^ *P*<0.01 *vs*. (-)CLA 5 μmol/L; ^&^ *P*<0.05 *vs*. (-)CLA 5 μmol/L; ^@@^ *P*<0.01 *vs*. (+)CLA 5 μmol/L.

The vinblastine-resistant KB cell line (KBv) expresses P-gp protein at a high level, which can be seen from verapamil inhibition with the same concentration 200μmol/L, leading to about 2-fold higher accumulation of Rh123 than that in Caco-2 and RBMECs(Figs 1–3); thus, the concentrations of CLA enantiomers were increased to 5 and 10 μmol/L. With lower concentrations, stereoselective differences in the regulation of P-gp activity between the (−) and (+)enantiomers were observed. The (+)enantiomer significantly decreased Rh123 intracellular accumulation by 25.9% (*P* < 0.01 *vs*. control), but the (−)enantiomer slightly increased intracellular Rh123 by 8.2% (Fig 3A). Similarly, a low concentration of (+)CLA antagonized the inhibition of verapamil to P-gp by 35.5% relative to verapamil treatment alone (Fig 3B). With a higher concentration, (+)CLA stimulation was altered to inhibition of P-gp and increased Rh123 intracellular accumulation by 24.5%, nearly the same as (−)CLA (30.0%; Fig 3B). Meanwhile, a synergistic effect on P-gp efflux activity was observed when (−) or (+)CLA was combined with verapamil (Fig 3B).

The stereoselective regulation of P-gp activity by CLA enantiomers in three cells that highly express P-gp protein is summarized in Table 1.

**Table 1:** Percentage of rhodamine 123 accumulation in Caco-2, KBv and RBMECs incubated with (−) or (+)CLA at two doses, relative to vehicle treatment.

Cell	Low dose		High dose	
	(−)CLA	(+)CLA	(−)CLA	(+)CLA
**Caco-2**	28.51	-11.68	62.97	58.22
**RBMEC**	20.04	-20.04	58.46	41.13
**KBv**	8.16	-25.92	30.00	24.49
Mean±SD, n = 3	18.90±10.22	-19.21±7.16	50.48±17.88	41.28±16.86

### Effects of CLA enantiomers on the P-gp expression and Rh123 accumulation in non-induced KB cells

**Fig 4:**
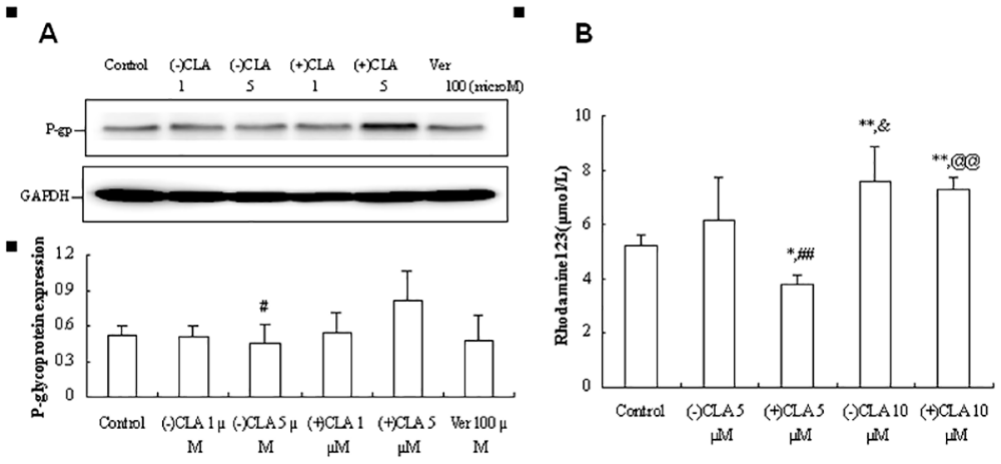
Effects of clausenamide(CLA) enantiomers on the P-gp expression(A) and the accumulation of rhodamine 123(B) in KB cells. Data were presented as Mean±SD from 3–5 independent experiments; (-) or (+)CLA: 1, or 5, or 10 μmol/L; verapamil(Ver): 100 μmol/L; rhodamine123: 5 μmol/L; *P<0.05, **P<0.01 *vs*. Control; ^#^ *P*<0.05, ^##^ *P*<0.01 *vs*. (-)CLA 5μmol/L; ^&^ *P*<0.05 *vs*. (-)CLA 5μmol/L; ^@@^ *P*<0.01 *vs*. (+)CLA 5μmol/L.

The P-gp expression in sensitive KB cells occurred at a very low level because of no induction of vinblastine at 200 nmol/L[19], which resulted in 5.8-fold higher intracellular fluorescence intensity (40.7 ± 7.2 a.u./mL/mg protein, n = 7) in KB cells than that in KBv cells (7.0 ± 3.2 a.u./mL/mg protein, n = 4) after treatment of Rh123 at 5 μmol/L for 2 h (in fact, the experiments in KB and KBv cells were conducted simultaneously). The P-gp expression in KB cells induced by (−) or (+)-isomer with the same concentrations which were applied to Caco-2 cells and RBMECs was investigated using Western blot analysis. As shown in Fig 4A, the P-gp expression was downregulated by (−)CLA (5 μmol/L) or verapamil (100 μmol/L) at 15.9% and 10.1%, respectively, but was upregulated by (+)CLA (5 μmol/L) at 54.5% relative to vehicle control, and there was a significant difference between the two enantiomers (*P* < 0.05). Meanwhile, the Rh123 accumulation in KB cells was increased by (−)CLA (5 μmol/L) at 18.0% and was decreased by (+)CLA at 28.2% (*P* < 0.05 *vs*. control), showing again significantly stereoselective differences between CLA enantiomers in P-gp-mediated transport of Rh123 (*P* < 0.01; Fig 4B).

### Effects of CLA concentrations on P-gp activity in Caco-2 cells

**Fig 5:**
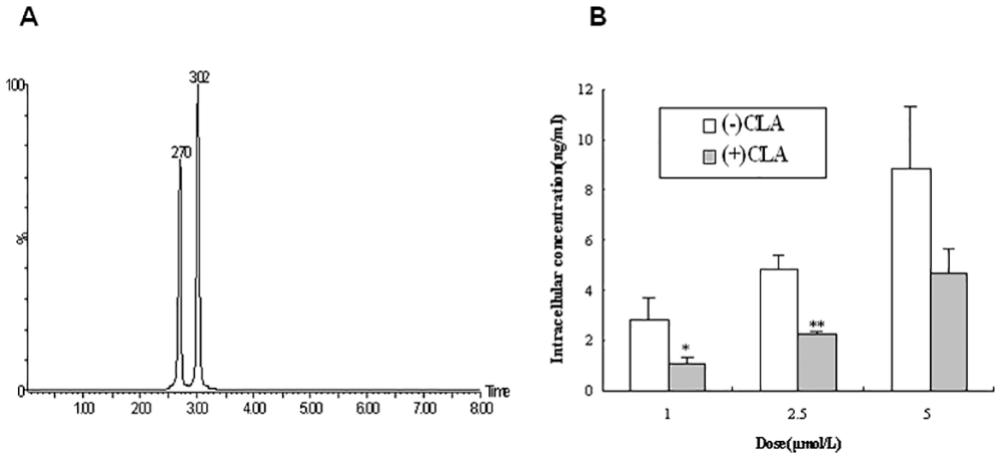
Chromatograms of clausenamide(CLA) and glipizide(IS) with retention time of 2.70 and 3.02min(A), and intracellular concentrations in Caco-2 cells treated with (-) or (+)CLA(B). Data were presented as Mean±SD from 3 independent experiments. (-) or (+)CLA: 1, 2.5 and 5 μmol/L; *P<0.05,**P<0.01 *vs*. (-)CLA.

To further elucidate the relations between CLA concentrations and P-gp activity and confirm the biphasic effects of the (+)-isomer, the uptake of (−) or (+)CLA in Caco-2 cells was detected by LC-MS/MS. As shown in Fig 5A, the separation of CLA enantiomers and glipizide (IS) with corresponding retention times of 2.70 min and 3.02 min was obtained. The sensitivity of this analytical method was 0.25 ng/mL, which was applicable for the determination of intracellular concentrations of CLA enantiomer. After treatment of Caco-2 cells with (−) or (+)CLA at 1 μmol/L for 2 h, the intracellular concentrations of the (−)-isomer were significantly greater than those of the (+)-isomer with the corresponding ratio [(−)/(+)] of 2.593 (*P* < 0.05) probably by the virtue of their different regulation of P-gp activity. When the concentrations of CLA were changed from 1 to 2.5 and 5 μmol/L, intracellular concentrations of (−)CLA increased in a concentration-dependent manner up to 8.853 ± 2.505 (ng/ml); meanwhile, the uptake of (+)CLA in Caco-2 cells also increased to 4.677 ± 1.029 (ng/mL); and their ratio [(−)/(+)] decreased to 1.893, indicating that the stimulatory effects of (+)CLA could be converted into inhibitory effects on P-gp activity (Table 2, Fig 5B).

**Table 2:** Uptake of clausenamide(CLA) enantiomers in Caco-2 cells(Mean±SD, n = 3).

Dose of CLA enantiomer (μmol/L)	Intracellular concentration(ng/ml)		(-)/(+)
	(−)CLA	(+)CLA	
1.0	2.834±0.808*	1.093±0.233	2.593
2.5	4.829±0.603**	2.228±0.065	2.167
5.0	8.853±2.505	4.677±1.029	1.893

**P<0.01,

*P<0.05 *vs*. (+)CLA by *t*-test.

## Discussion

In this study, three types of cells highly expressing P-gp, Caco-2, KBv and RBMEC, were used to investigate the effects of CLA enantiomers on the intracellular accumulation of Rh123 and to evaluate the regulation of CLA enantiomers on P-gp activity. The results from these cells showed that (−)CLA at a lower concentration (*e*.*g*. 1 μmol/L) increased Rh123 intracellular accumulation by 8.2%–28.5%, whereas (+)CLA reduced the accumulation by 11.7%–25.9%, showing significantly stereoselective differences between the two enantiomers in the regulation of P-gp-mediated Rh123 transport (Table 1). Furthermore, following co-treatment of these cells with each CLA enantiomer and verapamil as a P-gp inhibitor [3, 5, 7, 22, 23], the (+)-isomer clearly antagonized the inhibitory effects of verapamil on P-gp efflux, whereas the (−)-isomer had no antagonistic effects but rather slightly synergistic or additive effects(Figs 1–3). These results suggested that (−)CLA could be a P-gp inhibitor and (+)CLA could be an inducer of P-gp. Cetirizine, a second-generation non-sedating anti-histamine, had similar efficacy. When Caco-2 cells were pre-treated with 100 μmol/L of each cetirizine enantiomer, the efflux of Rh123 and doxorubicin was significantly enhanced by R-cetirizine but was reduced by S-cetirizine, suggesting that R-cetirizine upregulated P-gp expression but S-cetirizine downregulated P-gp expression[24]. It was also just as CLA enantiomers regulated the expression of P-gp and the P-gp-mediated accumulation of Rh123 in KB cells(Fig 4).

As higher concentrations (*e*.*g*. 5 μmol/L) of CLA enantiomers were added to these cells, the inhibitory effects of the (−)-isomer on P-gp further increased, resulting in enhanced Rh123 intracellular concentration by 30.0%–63.0%; whereas the stimulatory effects of the (+)-isomer were converted into inhibitory ones, also leading to an increase in the intracellular concentration of Rh123 by 24.5%–58.2%(Table 1), which indicated that (+)CLA could be a modulator of P-gp. Progesterone also showed such biphasic trends in P-gp-expressing cells. A low concentration of progesterone (6.3 μmol/L) stimulated P-gp ATPase activity, but at concentrations above 25 μmol/L, progesterone inhibited it[22], resulting in reduced hydrolysis of ATP and an enhanced P-gp efflux. Similarly, the relationship of different concentrations of (+)CLA with P-gp ATPase activity will be further investigated in these cells.

In addition, the biphasic effects of (+)CLA were confirmed in Caco-2 cells. Following a low concentration of (−) or (+)CLA (1 μmol/L), there were significant differences in their intracellular concentrations with a ratio [(−)/(+)] of 2.593 (*P* < 0.05) because of their inhibitory or stimulatory effects on P-gp activity, respectively. When a higher concentration (5 μmol/L) was applied, both (−) and (+)CLA showed inhibitory effects on P-gp, leading to an increase in intracellular uptake, and consequently a decrease in the ratios [(−)/(+)] to 1.893(Table 2, Fig 5); for (+)CLA, it could not result from saturation of P-gp activity since its intracellular concentrations were further increased and the same ratio was decreased to 1.091 in the presence of cyclosporine A (Data not shown). These results indicated once again that (+)CLA could be a modulator with concentration-dependent biphasic effects on P-gp efflux activity.

P-gp is expressed in normal tissues with excretory function (small intestine, liver and kidney), promoting drug elimination into faeces, bile, and urine, and at blood—tissue barriers (blood—brain barrier, blood—testis barrier and placenta), protecting various tissues from potentially toxic xenobiotics[25–28]. In the present study, the different effects of (−) or (+)CLA on P-gp activity in RBMECs, a main component of the *in vitro* model of the blood—brain barrier, provided a possible explanation for the previous findings that the amount of the (−)-isomer that entered into rat brain was significantly greater than that of the (+)-isomer 15 min after oral administration (*P* < 0.001)[18]. Moreover, the cumulative amounts of biliary and urinary excretion of the (+)-isomer were more than those of the (−)-isomer, with ratios [(+)/(−)] of 3.49 and 3.81, respectively[17], which could contribute to the stimulation of (+)CLA and the inhibition of (−)CLA on P-gp efflux in liver hepatocytes and proximal tubules of the kidney. P-gp-mediated biliary excretion of the (+)-isomer elicited a secondary peak in the elimination phase of the concentration—time curve in the portal vein of rabbits via hepato-enteric circulation, partially leading to its higher plasma concentrations compared with the (−)-isomer in the pre-systemic circulation[17]. Meanwhile, CYP3A was another contributor to the differences between the two enantiomers in plasma concentrations. CYP3A is expressed in the liver (the centrolobular regions near the central vein), gut (the brush border cells of the villi) and brain (hippocampus, hypothalamus, cerebellum and olfactory bulb)[29,30], whose substrates extensively overlap with P-gp[31–33]. Previous studies in our laboratory demonstrated that CYP3A1/2 was a major metabolizing enzyme of CLA enantiomers in rats, and the (−)-isomer could induce a higher expression of CYP3A1 than the (+)-isomer in the rat liver[17,34]. Thus, among the interactions of both proteins with (−) or (+)CLA at higher oral doses (*e*.*g*. a single dose of 160 mg/kg for rats), CYP3A may play a more important role than P-gp in the absorption and metabolic elimination of CLA enantiomers, which explains their stereoselective differences in three target tissues of the brain and plasma concentrations at 8 h and 12 h after dosing respectively[16,18], and provides useful information to the clinical pharmacology of (−)CLA as a candidate drug for treatment of Alzheimer’s disease.

## Conclusions

Consistent results from three cells expressing P-gp have demonstrated that (−)CLA could be a P-gp inhibitor, and the (+)CLA could be a modulator with concentration-dependent biphasic effects on P-gp activity, which may cause drug—drug interactions when combined with other P-gp substrate drugs.

## Supporting Information

S1 Appendix Primary cultures of rat brain microvessel endothelial cells. (DOC)
