# Supplementary material for: Stereoselective Regulation of P-gp Activity by Clausenamide Enantiomers in Caco-2, KB/KBv and Brain Microvessel Endothelial Cells
Source: PLoS One. 2015 Aug 21;10(8):e0135866. doi: 10.1371/journal.pone.0135866 (PMC4546427; doi:10.1371/journal.pone.0135866)
Supplement: S1 Appendix — (DOC) [file pone.0135866.s001.doc]

**Supporting Information**

Primary cultures of brain microvessel endothelial cells were prepared from Sprague–Dawley rats (10–12 days old; Beijing Vital River Company, Beijing, China), as previously described by Qian *et al.*[21]. Animals were anesthetized by intraperitoneal injection of 20% ethyl carbamate at dose of 1g/kg[35], and sterilized by immersion in 75% alcohol solution for 2 min. The gray matter of these animals was taken out and minced into small pieces of approximately 1 mm^3^ in ice-cold MEM medium, then dissociated by using 2 mg/mL collagenase containing DNase at a final concentration of 10.5 μg/ml on a shaker for 20 min at 37°C. The cells were pelleted by centrifugation(1000 rpm, 10 min, 4°C) and digested once again according to the above protocol. Then 20% dextran(Mr 40,000) was added to the cells for differential centrifugation at 2000 rpm for 10 min. The cells were re-suspended in complete MEM medium and passed through 74-μm stainless steel mesh. Obtained RBMECs were seeded on 1% (w/v) gelatin-coated culture flasks and cultivated in complete medium (MEM, 20% FBS, 0.9 mg/mL glutamate, 10 mmol/L hepes, 100 ng/mL bFGF, 100 μg/mL heparin sulphate, 100 units/mL penicillin, 100 μg/mL streptomycin sulphate and 50 units/mL amphotericin B) at 37°C and 5% CO_2_, which was refreshed with complete medium 4 h after seeding to eliminate non-endothelial cells. A final concentration of 2 μg/mL of puromycin was added for 7 h to further purify the cultured cells. The purity of RBMECs was examined by their morphological characteristics using a phase-contrast microscope, and immunocytochemistry of a specific marker, factor VIII-associated antigen, with a rabbit anti-factor VIII polyclonal antibody and FITC-conjugated goat anti-rabbit factor VIII secondary antibody by immunofluorescence microscopy.

**References**

21. Qian ZY, Huang Q, Zhou LY, Sun ZF. Isolation and long-term cultivation of rat brain microvascular endothelial cells. Chinese J Cell Biol 1999; 21:42-45.

35. Zhu CJ, Zhang JT. Effects of (−), (+)-7-hydroxy-clausenamide on synaptic transmission in rat dentate gyrus *in vitro*. Acta Pharm Sin 2004;39:34-36.
